# Long-term cultivation of retinal pigment epithelium cells on nanofiber scaffolds

**DOI:** 10.1007/s00417-024-06707-3

**Published:** 2025-01-15

**Authors:** Julian A. Zimmermann, Lucy Irlenbusch, Uwe Hansen, Marcus Himmler, Chun Zeng, Nicole Eter, Thomas Fuchsluger, Peter Heiduschka

**Affiliations:** 1 Department of Ophthalmology, University Hospital Munster, Munster, Germany; 2 Institute of Musculoskeletal Medicine, University Hospital Munster, Munster, Germany; 3 Institute of Polymer Materials, Friedrich-Alexander University Erlangen-Nuremberg, Erlangen, Germany; 4 Department of Ophthalmology, University Medical Center Rostock, Rostock, Germany

**Keywords:** Porcine retinal pigment epithelium, Nanofiber scaffolds, Macular degeneration, Cell culture, Cell replacement

## Abstract

**Purpose:**

The retinal pigment epithelium (RPE) plays an important role in the pathogenesis of age-related macular degeneration (AMD) and other retinal degenerative diseases. The introduction of healthy RPE cell cultures into the subretinal space offers a potential treatment strategy. The aim of this study was the long-term culture and characterisation of RPE cells on nanofiber scaffolds.

**Methods:**

Nanofiber scaffolds consisting of polycaprolactone (PCL) and collagen were prepared by electrospinning. Porcine RPE cell cultures were maintained on PCL scaffolds, PCL-collagen scaffolds, and controls at the bottom of 24-well plates. Cell culture analysis was performed by immunohistochemistry, while the release of inflammatory cytokines IL-6, IL-8, TNF-α, and PDGF-β was measured by ELISA and multiplex assays. Ultrastructural features were examined by transmission electron microscopy.

**Results:**

The observation period averaged 42.7 weeks for controls, 38.7 weeks for PCL scaffold cultures, and 36.1 weeks for PCL-collagen scaffold cultures, with cell number and morphology remaining stable. TNF-α levels in the supernatants were minimal, IL-6 levels were consistently low, and IL-8 levels decreased from initially high to lower levels over time.

**Conclusion:**

RPE cells were stably cultured on nanofiber scaffolds for extended periods of time. The long-term physiological properties of RPE cells, including phagocytic ability and visual cycle enzyme activity, need to be further investigated before clinical application. In addition, controlling the expression of inflammatory mediators is a major challenge. Despite these hurdles, overcoming them is critical given the increasing prevalence of retinal degenerative diseases.

## Abstract (highlights)

****Key Messages**:**

*****What is known***:**

- Retinal pigment epithelium (RPE) cells play an important role in the pathogenesis of degenerative retinal diseases.
- One therapeutic approach is to transplant healthy RPE cells into the subretinal space. 
- Various nanofiber scaffolds have been used in the past to replace Bruch's membrane.

*****What is new***:**

- RPE cells can be stably cultured on polycaprolactone (PCL) and collagen nanofiber scaffolds for up to one year. 
- RPE cells show characteristic properties in terms of cell number, morphology and secretion behaviour on PCL/collagen nanofiber scaffolds.

## Introduction

The retinal pigment epithelium (RPE) is a monolayer of polarised, hexagonal pigmented cells located on Bruch’s membrane between the endothelium of the choriocapillaris and the retinal photoreceptors [1].

The RPE has important functions. These include phagocytosis of photoreceptor outer segments, transport of nutrients and metabolites between the choriocapillaris and the retina, recycling of the visual pigment and secretion of growth factors. With its tight junctions, the RPE is part of the blood-retinal barrier [2–4].

Accumulation of lipofuscin in RPE cells and thickening of Bruch’s membrane throughout life contribute to RPE dysfunction and the pathogenesis of several neurodegenerative eye diseases such as age-related macular degeneration (AMD) [5]. First described by Haab in 1885, AMD is the leading cause of progressive visual impairment in people over the age of 50 in developed countries. There are currently no curative treatments available. The morbidity of AMD is expected to increase [6].

Since dysfunctional RPE is thought to be a major cause of photoreceptor degeneration in AMD and other degenerative diseases, its replacement by healthy RPE has been discussed as a therapeutic strategy. In the past, the knowledge gained about RPE cells led to the idea of transplanting them to replace non-functional pigment epithelium and to prevent secondary loss of retinal photoreceptors and visual impairment [7, 8]. Approaches have ranged from subretinal injection of RPE cells to the use of RPE and Bruch’s membrane grafts, with varying degrees of success.

Another proposed approach to deliver RPE cells into the subretinal space is the use of temporary or permanent biocompatible scaffolds on which the RPE cells have been cultured as a monolayer.

Nanofibrous membranes have been explored as artificial Descemet’s membrane grafts, drug delivery devices or superficial wound dressings [9–17]. The versatile and practical method of electrospinning allows the preparation of nanofibers from many different polymers [18].

With a variety of different successful approaches to replace defective RPE, the aim of this work was to test biodegradable electrospun nanofiber scaffolds in vitro as carriers for long-term cultivation of primary porcine RPE cells and to characterize these cells on the scaffolds in vitro.

## Materials and methods

### Nanofibers

The electrospinning process is described in the literature, e.g [18–20]. Briefly, a polymer solution or melt is extruded into an electric field, where the repulsive forces of the charge carriers on the surface of the polymer jet cause extensive stretching of the filament. This leads to the formation of thin fibers with radial dimensions starting from a few tens of nanometers. The nanofibers can be collected on a flat collector with random orientation of their fibre axis.

In practice, the polymer solutions were stirred overnight. Prior to electrospinning, the polymer solutions were filled into a glass syringe with an 18-gauge stainless steel needle attached. The syringe was then placed in a syringe pump to ensure a constant flow rate of the polymer solution. A high voltage source was attached to the stainless steel needle and an electric field was established between the needle and the grounded collector plate. For ease of use, the collector plate is wrapped in household aluminium foil to facilitate the handling of the nanofibers membranes.

In this study, the nanofibers scaffolds were prepared based on polycaprolactone (PCL, Mw = 80 kg/mol, Sigma Aldrich, Saint Louis, MO, USA). It was further mixed with collagen (Symathese, Lyon, France). Solvent systems of formic acid and acetic acid (both Carl Roth GmbH + Co. KG, Karlsruhe, Germany) in the ratio of 7:3 and 90% diluted acetic acid, respectively, were used for the polymer solutions. The experimental details are shown in Table 1.

**Table 1:** Parameters for the fabrication of nanofiber scaffolds

Poly-capro-lactone (PCL)	Blend component	Mass of blend component	Concentration	Solvent System	Voltage	Distance*	Flow Rate
0.6 g	–	–	1.2 g/ 10 ml	Formic acid / acetic acid (7:3)	22 kV	17 cm	0.2 ml/h
0.4 g	Collagen	0.2 g	1.2 g/ 10 ml	90% acetic acid	15 kV	15 cm	1 ml/h

* distance between the needle and the grounded plate

### Preparation of RPE cells

Porcine eyes were processed within 2 to 3 h (h) after enucleation. Disinfection was performed with a povidone-iodine solution (Mundipharma, Germany).

After removal of the vitreous, the eyecup was filled with 37 °C warm DPBS containing 1% streptomycin/penicillin (Sigma-Aldrich, #P4333) and the retina was removed. The eyecup was then filled with trypsin/EDTA (0.25%, Gibco, #25200-056) and incubated at 37 °C in 7.2% CO2 for 30 min (min). The RPE cells were then extracted from the eyecup by pipetting the trypsin solution up and down until the trypsin/EDTA suspension was homogeneously dark brown to black. The trypsin solution containing RPE cells was added to a 50 ml Falcon tube prefilled with nutrient medium consisting of DMEM + GlutaMAX (Gibco, #31966-021), 10% porcine serum (Bio&Sell, #scw.se.500), 1% non-essential amino acids (NEA, Gibco, #11140-035), and 1% penicillin-streptomycin, and centrifuged three times at 800 rpm (117 g) for 5 min each. The resulting RPE cells were incubated in cell culture flasks (25 cm² growth area, Sarstedt, #83.3910.002) in 6 ml fresh culture medium at 37 °C and 7.2% CO2.

### Cultivation of the RPE cells

Nanofiber scaffolds were cut to appropriate size to fit into the wells of a 24-well plate (11 × 11 mm²), disinfected in ethanol, and soaked in DPBS with 1% penicillin-streptomycin for 24 h. The scaffolds were transferred to a 24-well plate (TPP, #92424) in 500 µl of 37 °C culture medium. Plastic rings were used to secure the scaffolds to the bottom of the wells.

After a stable RPE cell culture was achieved in the flasks, the RPE cells were removed from the flasks and a suspension of 500,000 cells in 500 µl of nutrient medium was added to the wells of a 24-well plate with an additional 500 µl of nutrient medium on top of the nanofiber scaffolds or in wells without scaffolds (controls). Incubation was performed at 37 °C and 7.2% CO2. The medium was changed every 4 days during the observation period. Supernatants were collected approximately monthly between medium changes.

A total of three experimental groups were established and followed throughout the experimental period:

1. cultures of RPE cells on the bottom of the wells of a 24-well plate (controls),
2. culture of RPE cells on polycaprolactone (PCL) nanofiber scaffolds, and.
3. culture of RPE cells on PCL and collagen nanofiber scaffolds. Digital images of all cultures were captured using a transmitted light microscope with a bright-field optics (EVOS™ FL Imaging System, Thermo Fisher). Five areas of each well were selected to count cells in the controls and on the respective nanofiber networks. The prominent nuclei of the RPE cells were used as a guide. The number of was averaged.

### Immunocytochemistry

For immunocytochemical staining, the nanofiber scaffold was first gently pulled onto a slide with the cell-colonized side up and washed with DPBS. The cells were then fixed with 4% paraformaldehyde solution for 15 min. The cells were washed and blocked with 0.3% Triton X-100 and 1% BSA in DPBS for 1 h at room temperature. Primary antibodies were diluted 1:100 (anti-ZO-1, Invitrogen, #61–7300) and 1:150 (anti-RPE-65, abcam, ab13826) in 1% BSA solution. Slides were incubated with primary antibodies overnight. Secondary antibodies were then diluted in 1% BSA/PBS solution and added: an AlexaFluor488-conjugated antibody (life technologies, A21206) diluted 1:200 to detect ZO-1 and an AlexaFluor594-conjugated antibody (ThermoFisher A-11032) diluted 1:600 to detect RPE65. After 1 h of incubation, the tissues were washed with DPBS. Finally, the tissues were incubated with DAPI solution diluted 1:300 in PBS for 10 min. Tissues were washed and coverslipped with Immu-Mount (Shandon, #9990402). Analysis was performed using an EVOS™ FL fluorescence microscope (AMG).

### ELISA and multiplex assay

Cell culture supernatants from each well of the corresponding test series and from each of the three groups were analyzed. Collection was performed within a five-week time window over the entire 55-week observation period. Data were obtained by averaging the individual values obtained during the intervals.

### ELISA 

To investigate the release of the inflammatory cytokines tumor necrosis factor-α (TNF-α) (Invitrogen, porcine TNF-α ELISA kit, catalog no. ES24RB) and interleukin-6 (IL-6) (Invitrogen, porcine IL-6 ELISA kit, catalog no. ESIL6) by the RPE cells, ELISA was performed using cell culture supernatants. Frozen samples were thawed and diluted 1:2 in assay diluent. 100 µl of the diluted samples were incubated per well of the ELISA plate overnight at 4˚C. 100 µl of biotin conjugate was added and incubated for 1 h at room temperature. 100 µl streptavidin-HRP was added and incubated for 30 min. 100 µl TMB solution was added and incubated for 15 min. Finally, 50 µl stop reagent was added. Absorbance at 450 nm was measured using a Synergy H1 plate reader (Biotek). Cytokine concentrations were calculated from simultaneously recorded standard curves.

**Fig. 1:**
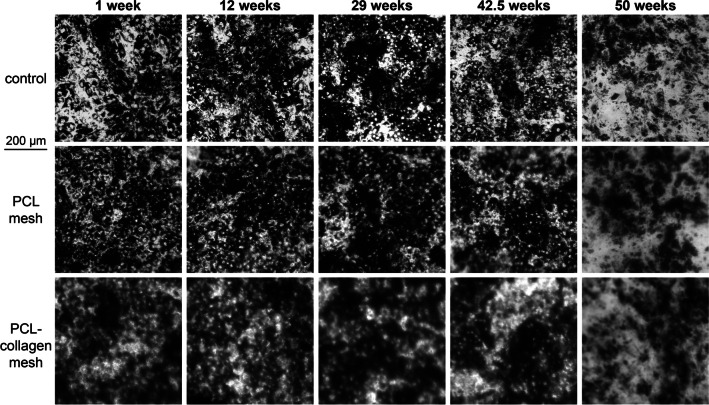
Examples of RPE cells cultured on the bottom of a 24-well plate (control), on a PCL nanofiber scaffold and on a PCL-collagen scaffold as indicated. The duration of culture is indicated at the top. The number of RPE cells and their pigmentation remain relatively stable over a long period of time. After 50 weeks of culture, the RPE cells were significantly depleted. Because the nanofiber scaffolds were not perfectly flat, some regions of the images are blurred

To determine the detection limits S_d_, we calculated them for all four analytes using the formula S_d_ = S_reag_ + 3×σ_reag_, where S_reag_ is the mean value of the signal for a reagent blank measured multiple times, and σ_reag_ is the known standard deviation for the signal of the reagent blank. We added a dashed line to each diagram in Fig. 1 to indicate the level of the corresponding detection limit.

### Multiplex assay

The concentrations of the inflammatory cytokines IL-6, IL-8, TNF-α and the platelet-derived growth factor β (PDGF-β) in the supernatants of the RPE cell cultures were determined using a Luminex multiplex assay (Luminex Discovery Assay, Porcine Premixed Multi-Analyte Kit, catalogue number: LXSAPM) according to the manufacturer’s instructions. Frozen samples were thawed, centrifuged and diluted 1:2 in the supplied diluent. 50 µl of each sample and standard were added to one well of the microtiter plate. 50 µl of a pre-diluted microparticle cocktail was added. The plate was then covered with foil, incubated for 2 h on a laboratory shaker at 800 rpm and subsequently stored overnight at 4˚C. The solution in the wells was discarded the next day. 50 µl of biotinylated antibody solution was added, and the plate was incubated for 1 h on an orbital shaker. 50 µl streptavidin-phycoerythrin solution was added and the plate was incubated on the shaker for 30 min. Finally, 100 µl of wash buffer was added to each well, and the plate was incubated on the shaker for 2 min. The microtiter plate was then read using a LUMINEX analyzer.

### Electron microscopy

Transmission electron microscopy (TEM) was used to characterize the ultrastructure of RPE cells. RPE cells on nanofiber scaffolds were fixed in 2% (v/v) formaldehyde and 2.5% (v/v) glutaraldehyde in 100 mM cacodylate buffer, pH 7.4, at 4 °C overnight. After washing in PBS, samples were postfixed in 0.5% (v/v) osmium tetroxide and 1% (w/v) potassium hexacyanoferrate(III) in 0.1 M cacodylate buffer for 2 h at 4 °C, followed by washing. After dehydration in an ascending ethanol series from 30 to 100% ethanol, samples were incubated twice for 15 min each in propylene oxide and embedded in Epon. Ultrathin sections were cut with an ultramicrotome, collected on copper grids, and negatively stained with 2% uranyl acetate for 10 min. Electron micrographs were taken at 60 kV with a Phillips EM-410 electron microscope using imaging plates (Ditabis, Pforzheim, Germany).

### Statistical evaluation

The GraphPad™ Prism 10 software (GraphPad Software Inc., La Jolla, CA, USA) was used to perform statistical calculations and to generate graphs. Significance levels were calculated using the non-parametric Kruskal-Wallis test and Dunn’s multiple comparison test. Significance was considered to be at *p* < 0.05.

## Results

RPE cells could be stably cultured on the polycaprolactone (PCL) and PCL-collagen nanofiber scaffolds. In total, we started nine runs under control conditions, nine runs on PCL scaffolds, and seven runs on PCL-collagen scaffolds. Six runs were included in the further evaluation. They lasted from 32.7 to 54.7 weeks, or approximately 8 to 12 months. The average duration of runs 2 through 9 was 42.7 weeks for controls (32.7–54.7 weeks), 38.7 weeks for cells on PCL scaffold (12.1–54.7 weeks), and 36.1 weeks for PCL-collagen scaffold (12.1–49.3 weeks). The experiments were terminated when the RPE cells showed clear signs of deterioration.

**Fig. 2:**
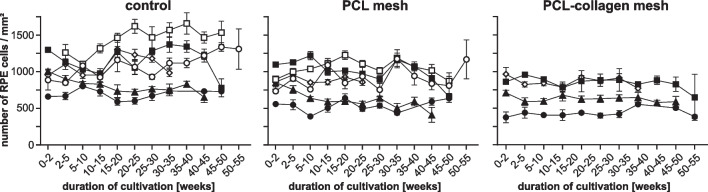
Number of RPE cells per mm² in cultures at the bottom of one well of a 24-well plate (control), on PCL scaffolds and on PCL-collagen scaffolds as indicated. Data were obtained by averaging the pooled values obtained during the time intervals indicated on the x-axis. Average values ± standard error of mean are shown. The symbols refer to the cultivation runs performed: • run 2, ○ run 3, ■ run 4, □ run 5, ▲ run 6, ◇ run 9

Examples of RPE cell appearance are shown in Fig. 2 for both control conditions and on a PCL nanofiber scaffold. The RPE cell layer was stabilized two days after seeding. Throughout the culture period, the cells maintained their pigmentation and did not show any significant changes over the next few months until the above-mentioned degradation began, i.e., their integrity and number were reduced and they showed a transition to fibroblast-like cells.

**Fig. 3:**
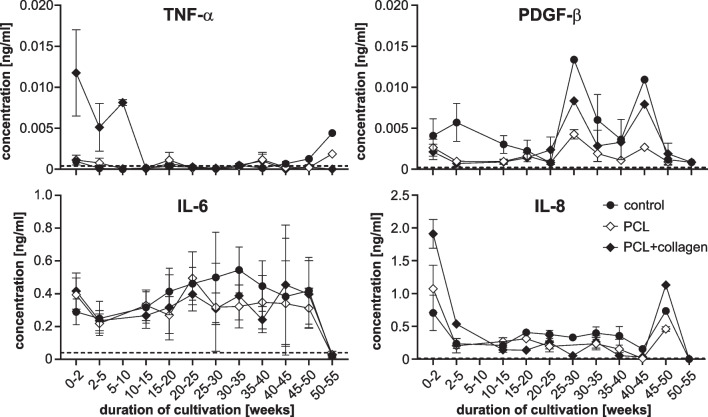
Concentrations of TNF-a, PDGF-b, IL-6 and IL-8 in cell culture supernatants as indicated. Data were obtained by averaging the values obtained during the intervals indicated on the x-axis. Error bars are standard errors of the mean. Note the different scales. Symbols indicate different culture conditions: • cells under control conditions,♢ cells on PCL scaffolds, and ♦ cells on PCL-collagen scaffolds

The density of cultured cells was determined by counting cells in digital images. Values obtained from images taken within the first week of culture were used as baseline values, and RPE cell density was monitored throughout the culture period. The results are shown in Fig. 3.

As shown in Fig. 3, the median cell densities were as follows:

- controls: 978 cells/mm² (range 600 to 1299 cells/mm²)
- on the PCL scaffolds: 855 cells/mm² (range 552 to 1097 cells/mm²), andon the PCL-collagen scaffolds: 768 cells/mm² (range 401 to 964 cells/mm²).
- on the PCL-collagen scaffolds: 768 cells/mm² (range 401 to 964 cells/mm²).

The highest number of cells was observed on the bottom of the 24-well plates under control conditions, and the lowest number of RPE cells was observed on the PCL-collagen scaffolds.

We also examined the release of the pro-inflammatory cytokines TNF-α, IL-6 and IL-8 and the growth factor PDGF-β by analysing the cell culture supernatants by ELISA and a multiplex assay. Since the results were in the same range, we pooled them. The results of the concentration measurements are shown in Fig. 1.

The four proteins analysed in Fig. 1 show different behaviours over time. The concentration of TNF-α in the supernatants was close to zero in most cases, except for the first weeks with the cells on the PCL-collagen scaffolds and the last weeks for the cells under control conditions and on the PCL scaffolds. PDGF-β levels were low during the first four months and later showed some irregular behaviour. The concentration of IL-6 remained at a relatively constant level throughout the observation period and approached zero at the end. IL-8 concentration decreased from a higher level to a lower level and increased just before the end of the observation period, only to decrease to zero at the end.

**Fig. 4:**
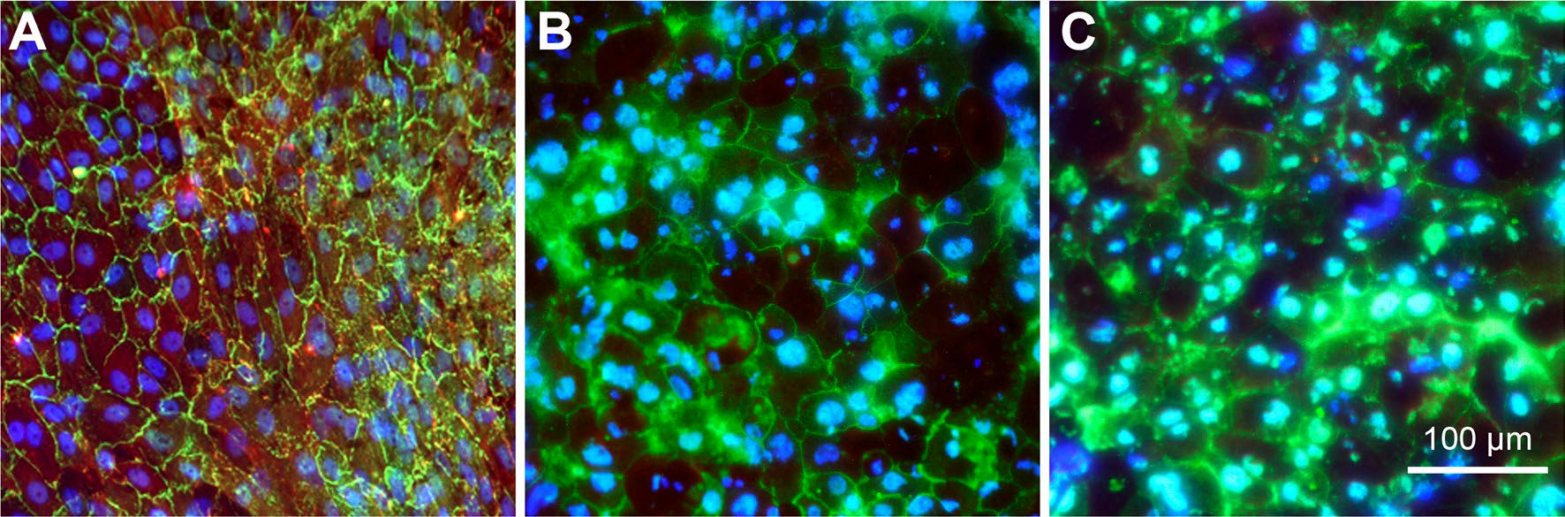
Immunocytochemical staining of RPE cells on the bottom of a 24-well plate (control) (**A**), on a PCL scaffold (**B**), and on a PCL-collagen scaffold (**C**) for RPE65 (red) and ZO-1 (green). Nuclei were stained with DAPI (blue). Cultivation time: (**A**) 10 days, (**B**) and (**C**) one month

Immunocytochemical staining of cultured RPE cells partially showed areas where the cell density was high and stable enough to form tight junctions, which could be identified by their immunoreactivity for ZO-1, as shown in Fig. 4A, after 10 days of cultivation under control conditions (Fig. 4A). When RPE cells were cultivated on the nanofiber scaffolds, we also found areas with a confluent RPE cell layer and ZO-1 immunoreactivity at the cell-cell contacts (Fig. 4B and C). However, we also saw a diffuse intracellular immunoreactivity for ZO-1 in a number of cells that were cultivated on the scaffolds.

Although the identity of the cultured cells was beyond doubt due to their strong pigmentation, we also performed staining for the RPE cell marker RPE65. While immunoreactivity for RPE65 was detected shortly after the start of the culture (Fig. 4A), it diminished later after a longer time of cultivation (Fig. 4B and C).

**Fig. 5:**
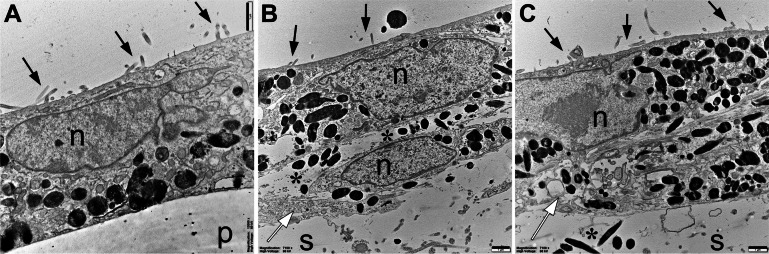
Transmission electron microscopy images of RPE cells on a plastic cell culture insert (control condition, **A**), on a PCL nanofiber scaffold (**B**), and on a PCL-collagen scaffold (**C**), each after one month of culture. The side of the corresponding growth substrate is indicated by “p” for plastic in **A** and by “s” for scaffold in **B** and **C**. Black arrows indicate microvilli and white arrows indicate some damage to RPE cells. Black asterisks are placed near extracellular melanin granules

For electron microscopy imaging under control conditions, RPE cells were cultivated in cell culture inserts. The appearance of microvilli (black arrows) indicated polarization of RPE cells. On nanofiber scaffolds, RPE cells also showed microvilli (Fig. 5B and C), demonstrating polarization of RPE cells also on the scaffolds. In many places, we saw an RPE cell lying at least partially overlying another RPE cell (Fig. 5B and C). In such cases, the lower cells showed some signs of damage (white arrows), and melanin granula lying outside of the RPE cells (black asterisks). No nanofibers of the scaffold could be identified in the images, most likely because they have been dissolved during the process of embedding into the Epon matrix. 5).

## Discussion

Due to an aging population, degenerative retinal diseases will become increasingly important in the future [21]. In a number of these diseases, dysfunction and loss of retinal pigment epithelium (RPE) leads to a degeneration of retinal photoreceptors and consequent loss of vision in the affected areas. Therefore, replacement of defective or degenerated RPE with healthy RPE cells is considered an approach to treat these degenerative diseases. After decades of scientific work with RPE cell cultures and multiple strategies to treat RPE cell pathologies, there is still no established method to sustainably replace RPE cells in vivo.

Therapeutic trials using subretinal injection of RPE cell suspensions have shown some promising results. However, cell attachment, migration, and confluent monolayer formation in the recipient eye are not satisfactory. In addition, the underlying Bruch’s membrane (BM) may be the site of degenerative processes in the retina, particularly membrane thickening and deposition of extracellular material, phospholipids, and drusen.

The use of scaffolds as a flexible support for healthy RPE cells for the ordered delivery of RPE cells into the subretinal space in combination with the replacement of the thickened BM addresses these challenges.

A variety of different materials have been tested for culturing RPE cells of different origins.

Natural materials include membranes and explants such as the anterior lens capsule, Bruch’s membrane, amniotic membrane, and Descemet’s membrane, as well as collagen, fibrinogen, gelatin, and cellulose [22].

Nanofibrous scaffolds mimic the 3-dimensional structure of BM and can serve as a substrate for RPE cell culture [23]. Poly(lactic-co-glycolic acid) (PLGA), poly(l-lactic acid) (PLLA), poly(caprolactone) (PCL), polyethylene glycol dimethacrylate (PEGDMA), poly(trimethylene) carbonate (PTMC), and polydimethylsiloxane (PDMS) are examples of synthetic materials used for RPE cell transplantation [22].

Stanzel et al. presented the transplantation of adult human RPE stem cells on polyester membranes into the subretinal space of rabbits. Four weeks after the procedure, a polarized monolayer of RPE cells was found on the membrane [24].

In 2014, Liu et al. tested subretinal transplantation of human fetal RPE cells on a polyethylene terephthalate and poly(l-lactide-co-ε-caprolactone) scaffold in rabbits. The RPE cultures achieved dense and adherent monolayers with tight junction expression and physiological cell morphology. The working group sees this method as a future therapeutic approach for retinal diseases leading to blindness, if it is biocompatible [25].

In the present study, biodegradable electrospun nanofiber scaffolds made of PCL and PCL combined with collagen were evaluated for their suitability for long-term cultivation of RPE cells. In addition, the release of various cytokines and growth factors by the RPE cells was investigated.

RPE cell density in the human eye decreases from the center of the macula to the periphery. While there are about 4000 cells/mm² in the center, there are only about 1600 cells/mm² in the periphery in vivo. This means that our results for both controls and nanofiber scaffolds are below the values for the retinal periphery [26]. In a study with fetal human RPE cells cultured on porous and non-porous PCL and on polyester transwells, McHugh et al. achieved cell densities of approximately 2000–3500 cells/mm² over a period of eight weeks. The differences to our work are the origin of the cells and the use of transwells [27]. Cell counts in our study remained fairly constant in all three groups. At week 40, there was a tendency for the cell count to decrease (Fig. 2 + 2). Nevertheless, counting cells on nanofiber scaffolds is challenging, in our case especially on the PCL-collagen scaffolds. One reason is that the cells are densely packed both on the bottom of the 24-well plates (controls) and on the scaffolds, and another reason is that the cells were sometimes stacked on top of each other.

The PCL-collagen scaffolds used showed higher opacity compared to the controls and theo-PCL scaffolds.

In addition, the quality of the transmitted light images degrades over time as any physical manipulation results in dislocation of the fragile scaffolds in the individual wells.

The cells retained their characteristic dark pigmentation throughout the observation period (Fig. 2). This is noteworthy because loss of pigmentation is common in cell culture and is considered one of the major challenges in RPE cell culture [28]. To our knowledge, this is the longest RPE cell culture on PCL and PCL and collagen nanofiber scaffolds published to date.

Immunocytochemical staining showed that RPE cells formed a dense monolayer with a hexagonal morphology at many sites on the nanofiber scaffolds in our study. Tight junctions, representing epithelial barrier formation, were clearly visible using tight junction ZO-1 staining (Fig. 4).

Electron microscopy showed that RPE cells on the nanofiber scaffolds had some physiological characteristics of RPE cells observed in vivo; RPE cell nuclei and cellular organelles, including melanosomes, could be identified. We did not see the nanofibers of the scaffold, only numerous small holes (Fig. 5). Most likely, the PCL polymer was dissolved in the Epon resin during the embedding process. On the other side of the RPE cell layer, many small circular structures were seen, most likely cut RPE cell microvilli. A few of these were also seen on the scaffold side, but the vast majority of these structures are on the other side, presumably the apical side of the RPE cells. This suggests polarization of the cultured RPE cells, which is an in vivo hallmark of RPE cells [3].

The expression and secretion of inflammatory cytokines by RPE cells must be considered when testing materials for RPE implantation. Such cytokines may attract microglia to migrate into the subretinal space, leading to inflammatory responses and reduced survival of transplanted RPE cells [22, 25, 29]. RPE cells can also contribute to leukocyte activation by secreting chemokines such as interleukin-8 (IL-8) and interleukin-6 (IL-6). The production of IL-8 and IL-6 in RPE cells can be stimulated by the pro-inflammatory cytokine TNF-α [30, 31].

Our study shows a similar pattern of secretion of TNF-α and IL-8 over the observation period (Fig. 1). While the secretion of TNF-α and IL-8 on the PCL-collagen scaffolds was elevated during the first few weeks of incubation, the concentration subsequently decreased to low levels only to increase again toward the end of the observation period. This time point coincided with the overall decrease in cell integrity and density in all three study arms.

Under in vitro conditions, porcine RPE cells produce very low levels of IL-6 [32]. We found an IL-6 level that was significantly higher than the levels of TNF-α or PFGFβ, and that the IL-6 level increased slightly during the observation period. The reason for the difference to Dietrich et al. may be the different time period before the growth medium was collected for analysis - while they took the medium after 24 h, we waited three days. Benson et al. analyzed the release of IL-6 by human RPE cells and found a very low initial IL-6 release and a gradual accumulation of IL-6 in the supernatant with increasing time [3]. Thus, RPE cells appear to continuously release IL-6 at a low but constant level even in the absence of stimulating cytokines such as IL-1. It is likely that the prolonged culture of RPE cells in vitro, i.e. under artificial conditions, gradually leads to an increased production of IL-6.

A similar effect may be present for PDGF-β, which was found to be released mainly in the second half of the observation period (Fig. 1). Under in vivo conditions, RPE cells produce PDGF-β among other growth factors [3]. PDGF-β is involved in angiogenesis and wound healing. It is associated with proliferative vitreoretinal diseases such as proliferative vitreoretinopathy and AMD [34]. Under culture conditions, RPE cells produce only little PDGF-β if not stimulated [35, 36]. As shown in Fig. 1, a long-term culture can lead to an increase of PDGF-β release even in the absence of other stimuli.

A sharp decrease in the secretion of IL-6, IL-8 and PDGF-β was observed at the end of the observation period, coinciding with the de-differentiation of the RPE cells and their death in culture.

## Conclusion

While RPE cells have been cultured on PCL and PCL and collagen nanofiber scaffolds for up to 10 days in a previous study, we have now shown that such cultivation is also possible for longer periods of time. The stability of cell density, cell morphology, and growth factor secretion was also demonstrated over several months, indicating the potential suitability of the nanofiber scaffold as a substrate for RPE cell implantation. The physiological properties of RPE cells on the scaffolds over such a long period of time, such as the formation and maintenance of polarity, phagocytic capacity, and the presence and function of visual cycle enzymes, still need to be investigated before they are ready for application. In addition, the expression of inflammatory mediators remains a challenge. However, given the increasing prevalence of retinal degenerative diseases and the lack of established therapeutic approaches, these challenges are worth addressing.

## References

[1] Boulton M, Dayhaw-Barker P (2001) The role of the retinal pigment epithelium: topographical variation and ageing changes. Eye 15:384–389. 10.1038/eye.2001.141 11450762 10.1038/eye.2001.141

[2] Bok D (1993) The retinal pigment epithelium: a versatile partner in vision. J Cell Sci Suppl 17:189–195 8144697 10.1242/jcs.1993.supplement_17.27

[3] Strauss O (2005) The retinal pigment epithelium in visual function. Physiol Rev 85:845–881. 10.1152/physrev.00021.2004 15987797 10.1152/physrev.00021.2004

[4] Rodriguez de Turco EB, Parkins N, Ershov AV et al (1999) Selective retinal pigment epithelial cell lipid metabolism and remodeling conserves photoreceptor docosahexaenoic acid following phagocytosis. J Neurosci Res 57:479–486 10440897

[5] Feeney-Burns L, Hilderbrand ES, Eldridge S (1984) Aging human RPE: morphometric analysis of macular, equatorial, and peripheral cells. Invest Ophthalmol Vis Sci 25:195–200 6698741

[6] Klein R, Klein BE, Jensen SC et al (1997) The five-year incidence and progression of age-related maculopathy. Ophthalmology 104:7–21. 10.1016/S0161-6420(97)30368-6 9022098 10.1016/s0161-6420(97)30368-6

[7] Algvere PV, Gouras P, Dafgård Kopp E (1999) Long-term outcome of RPE allografts in non-immunosuppressed patients with AMD. Eur J Ophthalmol 9:217–230 10544978 10.1177/112067219900900310

[8] Li LX, Turner JE (1988) Inherited retinal dystrophy in the RCS rat: prevention of photoreceptor degeneration by pigment epithelial cell transplantation. Exp Eye Res 47:911–917 3215300 10.1016/0014-4835(88)90073-5

[9] Teichmann J, Valtink M, Nitschke M et al (2013) Tissue engineering of the corneal endothelium: a review of carrier materials. J Funct Biomater 4:178–208. 10.3390/jfb4040178 24956190 10.3390/jfb4040178 PMC4030930

[10] Kong B, Mi S (2016) Electrospun Scaffolds for Corneal Tissue Engineering: A Review. Mater (Basel) 9. 10.3390/ma9080614 10.3390/ma9080614 PMC5509008 28773745

[11] Kruse M, Walter P, Bauer B et al (2018) Electro-spun membranes as scaffolds for human corneal endothelial cells. Curr Eye Res 43:1–11. 10.1080/02713683.2017.1377258 29281419 10.1080/02713683.2017.1377258

[12] Himmler M, Garreis F, Paulsen F et al (2021) Optimization of polycaprolactone - based nanofiber matrices for the cultivation of corneal endothelial cells. Sci Rep 11:18858. 10.1038/s41598-021-98426-6 34552187 10.1038/s41598-021-98426-6 PMC8458296

[13] Himmler M, Schubert DW, Dähne L et al (2022) Electrospun PCL Scaffolds as Drug Carrier for Corneal Wound Dressing Using Layer-by-Layer Coating of Hyaluronic Acid and Heparin. Int J Mol Sci 23. 10.3390/ijms23052765 10.3390/ijms23052765 PMC8910869 35269908

[14] Himmler M, Schubert DW, Fuchsluger TA (2021) Examining the Transmission of Visible Light through Electrospun Nanofibrous PCL Scaffolds for Corneal Tissue Engineering. Nanomaterials (Basel) 11. 10.3390/nano11123191 10.3390/nano11123191 PMC8705195 34947541

[15] Küng F, Schubert DW, Stafiej P et al (2016) A novel suture retention test for scaffold strength characterization in ophthalmology. Mater Sci Eng C Mater Biol Appl 69:941–946. 10.1016/j.msec.2016.07.052 27612789 10.1016/j.msec.2016.07.052

[16] (2011) Electrospinning for tissue regeneration. Woodhead publishing in materials. Woodhead Pub, Oxford, Cambridge

[17] Küng F, Schubert DW, Stafiej P et al (2017) Influence of operating parameters on the suture retention test for scaffolds in ophthalmology. Mater Sci Eng C Mater Biol Appl 77:212–218. 10.1016/j.msec.2017.02.177 28532023 10.1016/j.msec.2017.02.177

[18] Wendorff JH (2012) Electrospinning: materials, processing, and applications. Wiley, Weinheim, Hoboken, N.J

[19] Schubert DW (2019) Revealing Novel Power laws and quantization in Electrospinning considering jet splitting—toward Predicting Fiber Diameter and its distribution. Macromol Theory Simul 28:1900006. 10.1002/mats.201900006

[20] Schubert DW, Allen V, Dippel U (2021) Revealing Novel Power laws and quantization in Electrospinning considering jet splitting—toward Predicting Fiber Diameter and its distribution part II experimental. Adv Eng Mater 23:2001161. 10.1002/adem.202001161

[21] Bindewald A, Roth F, van Meurs J et al (2004) Transplantation Von Retinalem Pigmentepithel (RPE) Nach CNV-Exzision bei altersabhängiger makuladegeneration. Techniken, Ergebnisse Und Perspektiven (transplantation of retinal pigment pithelium (RPE) following CNV removal in patients with AMD. Techniques, results, outlook). Ophthalmologe 101:886–894. 10.1007/s00347-004-1077-2 15316735 10.1007/s00347-004-1077-2

[22] White CE, Olabisi RM (2017) Scaffolds for retinal pigment epithelial cell transplantation in age-related macular degeneration. J Tissue Eng 8:2041731417720841. 10.1177/2041731417720841 28794849 10.1177/2041731417720841 PMC5524239

[23] Hotaling NA, Khristov V, Wan Q et al (2016) Nanofiber Scaffold-based tissue-Engineered Retinal Pigment Epithelium to treat degenerative Eye diseases. J Ocul Pharmacol Ther 32:272–285. 10.1089/jop.2015.0157 27110730 10.1089/jop.2015.0157 PMC4904235

[24] Stanzel BV, Liu Z, Somboonthanakij S et al (2014) Human RPE stem cells grown into polarized RPE monolayers on a polyester matrix are maintained after grafting into rabbit subretinal space. Stem Cell Rep 2:64–77. 10.1016/j.stemcr.2013.11.005 10.1016/j.stemcr.2013.11.005 PMC3916756 24511471

[25] Liu Z, Yu N, Holz FG et al (2014) Enhancement of retinal pigment epithelial culture characteristics and subretinal space tolerance of scaffolds with 200 nm fiber topography. Biomaterials 35:2837–2850. 10.1016/j.biomaterials.2013.12.069 24439407 10.1016/j.biomaterials.2013.12.069

[26] Panda-Jonas S, Jonas JB, Jakobczyk-Zmija M (1996) Retinal pigment epithelial cell count, distribution, and correlations in normal human eyes. Am J Ophthalmol 121:181–189 8623888 10.1016/s0002-9394(14)70583-5

[27] McHugh KJ, Tao SL, Saint-Geniez M (2014) Porous poly(ε-caprolactone) scaffolds for retinal pigment epithelium transplantation. Invest Ophthalmol Vis Sci 55:1754–1762. 10.1167/iovs.13-12833 24550370 10.1167/iovs.13-12833 PMC3968933

[28] Fronk AH, Vargis E (2016) Methods for culturing retinal pigment epithelial cells: a review of current protocols and future recommendations. J Tissue Eng 7. 10.1177/2041731416650838 10.1177/2041731416650838 PMC4959307 27493715

[29] White C, DiStefano T, Olabisi R (2017) The influence of substrate modulus on retinal pigment epithelial cells. J Biomed Mater Res A 105:1260–1266. 10.1002/jbm.a.35992 28028920 10.1002/jbm.a.35992 PMC6519294

[30] Elner VM, Burnstine MA, Strieter RM et al (1997) Cell-associated human retinal pigment epithelium interleukin-8 and monocyte chemotactic protein-1: immunochemical and in-situ hybridization analyses. Exp Eye Res 65:781–789. 10.1006/exer.1997.0380 9441701 10.1006/exer.1997.0380

[31] Bernardo-Colón A, Lerner M, Becerra SP (2022) Pigment epithelium-derived factor is an interleukin-6 antagonist in the RPE: insight of structure-function relationships. Front Physiol 13:1045613. 10.3389/fphys.2022.1045613 36467689 10.3389/fphys.2022.1045613 PMC9709256

[32] Dietrich L, Lucius R, Roider J et al (2020) Interaction of inflammatorily activated retinal pigment epithelium with retinal microglia and neuronal cells. Exp Eye Res 199:108167. 10.1016/j.exer.2020.108167 32735798 10.1016/j.exer.2020.108167

[33] Benson MT, Shepherd L, Rees RC et al (1992) Production of interleukin-6 by human retinal pigment epithelium in vitro and its regulation by other cytokines. Curr Eye Res 11:173–179. 10.3109/02713689208999529 1424742 10.3109/02713689208999529

[34] Nagineni CN, Kutty V, Detrick B et al (2005) Expression of PDGF and their receptors in human retinal pigment epithelial cells and fibroblasts: regulation by TGF-beta. J Cell Physiol 203:35–43. 10.1002/jcp.20213 15368539 10.1002/jcp.20213

[35] Handa JT, Reiser KM, Matsunaga H et al (1998) The advanced glycation endproduct pentosidine induces the expression of PDGF-B in human retinal pigment epithelial cells. Exp Eye Res 66:411–419. 10.1006/exer.1997.0442 9593635 10.1006/exer.1997.0442

[36] Kernt M, Neubauer AS, Liegl RG et al (2010) Sorafenib prevents human retinal pigment epithelium cells from light-induced overexpression of VEGF, PDGF and PlGF. Br J Ophthalmol 94:1533–1539. 10.1136/bjo.2010.182162 20962354 10.1136/bjo.2010.182162

